# Two Sides of the Same Coin: A Case Report of First-Episode Catatonic Syndrome in a High-Functioning Autism Patient

**DOI:** 10.3389/fpsyt.2019.00224

**Published:** 2019-04-12

**Authors:** Dimitri Hefter, Cristina E. Topor, Peter Gass, Dusan Hirjak

**Affiliations:** ^1^Department of Psychiatry and Psychotherapy, Central Institute of Mental Health, Medical Faculty Mannheim, University of Heidelberg, Mannheim, Germany; ^2^Institute of Physiology and Pathophysiology, University of Heidelberg, Heidelberg, Germany

**Keywords:** autism, catatonia, schizophrenia, diagnostic challenge, acute psychiatry, coercive treatment

## Abstract

**Background:** Catatonic phenomena such as stupor, mutism, stereotypy, echolalia, echopraxia, affective flattening, psychomotor deficits, and social withdrawal are characteristic symptoms of both schizophrenia and autism spectrum disorders (ASD), suggesting overlapping pathophysiological similarities such as altered glutamatergic and dopaminergic synaptic transmission and common genetic mutations. In daily clinical practice, ASD can be masked by manifest catatonic or psychotic symptoms and represent a diagnostic challenge, especially in patients with unknown or empty medical history. Unclear diagnosis is one of the main factors for delayed treatment. However, we are still missing diagnostic recommendations when dealing with ASD patients suffering from catatonic syndrome.

**Case presentation:** A 31-year-old male patient without history of psychiatric disease presented with a severe catatonic syndrome and was admitted to our closed psychiatric ward. After the treatment with high-dose lorazepam and intramuscular olanzapine, catatonic symptoms largely remitted, but autistic traits persisted. Following a detailed anamnesis and a thorough neuropsychological testing, we diagnosed the patient with high-functioning autism and catatonic schizophrenia. The patient was discharged in a remitted state with long-acting injectable olanzapine.

**Conclusion:** This case represents an example of diagnostic and therapeutic challenges of catatonic schizophrenia in high-functioning autism due to clinical and neurobiological overlaps of these conditions. We discuss clinical features together with pathophysiological concepts of both conditions. Furthermore, we tackle social and legal hurdles in Germany that naturally arise in these patients. Finally, we present diagnostic “red flags” that can be used to rationally select and conduct current recommended diagnostic assessments if there is a suspicion of ASD in patients with catatonic syndrome in order to provide them with the most appropriate treatment.

## Background

Schizophrenia and autism spectrum disorders (ASD) are common, highly heritable, and severe neurodevelopmental disorders with overlapping genetic, neurobiological, and clinical characteristics. Schizophrenia is a complex brain disorder with an average prevalence of about 0.6% (1). ASD are classified as pervasive developmental disorders in the *Diagnostic and Statistical Manual of Mental Disorders, Fifth Edition* (DSM-5) with an estimated prevalence of 0.6–1% in the general population (2). The core symptoms of ASD are impairments in social interaction, impairments in communication, and restricted as well as repetitive behaviors (3). ASD are usually diagnosed in childhood or adolescence. If the patient exhibits flamboyant psychotic and catatonic symptoms, clinicians are confronted with considerable difficulties in diagnosing both disorders. Due to potentially life-threatening consequences, fast diagnostic workup is required in order to provide correct treatment.

From a clinical perspective, catatonic phenomena are nosologically unspecific and involve complex affective, motor, and behavioral symptoms (4, 5). According to International Classification of Diseases (ICD-10), catatonia can only be diagnosed within the group of schizophrenia spectrum disorders or within the organic catatonic disorder. However, the majority of studies in recent years have made no distinction between the different forms of schizophrenia (6). The new classification systems DSM-5 and International Classification of Diseases (ICD-11) have also abandoned the traditional differentiation into paranoid, catatonic, hebephrenic, and disorganized subtype. DSM-5 classifies catatonia as a specifier in order to characterize other mental disorders presenting with catatonic features. Catatonia in the ICD-11 (https://icd.who.int/dev11) will be listed as an independent entity belonging to the mental, behavioral, or neurodevelopmental disorders. However, such discrepancies in the definition of catatonic syndrome might confound the diagnosis and delay appropriate treatment of patients presenting with severe catatonia. In this case report, we will discuss current evidence-based practice guidelines for accurate assessment, treatment, and management of catatonic syndrome in adult patients with high-functioning ASD.

## Case Presentation and Clinical Assessment

A 31-year-old Caucasian male patient with catatonia was admitted to our closed psychiatric ward. In the emergency contact, he was disoriented as to the situation, time, and place; confused; anxious; and mutistic. Besides incoherently expressed psychotic fears of poisoning and other incoherent phrases, he was not open for exploration. Most information was gathered from his accompanying parents. According to them, he had never moved out but had been living his whole adult life in the basement of their house. Usually socially withdrawn and very calm, his behavior had changed rapidly approximately 5 days prior to admission toward agitation, repetitive movements, verbal and physical aggression, and sexual disinhibition. The patient had no prior personal or family psychiatric history and no history of drug abuse. Besides being underweight (Body Mass Index = 18.4 kg/m^2^), he was in good physical health and had never taken medication.

**Figure 1:**
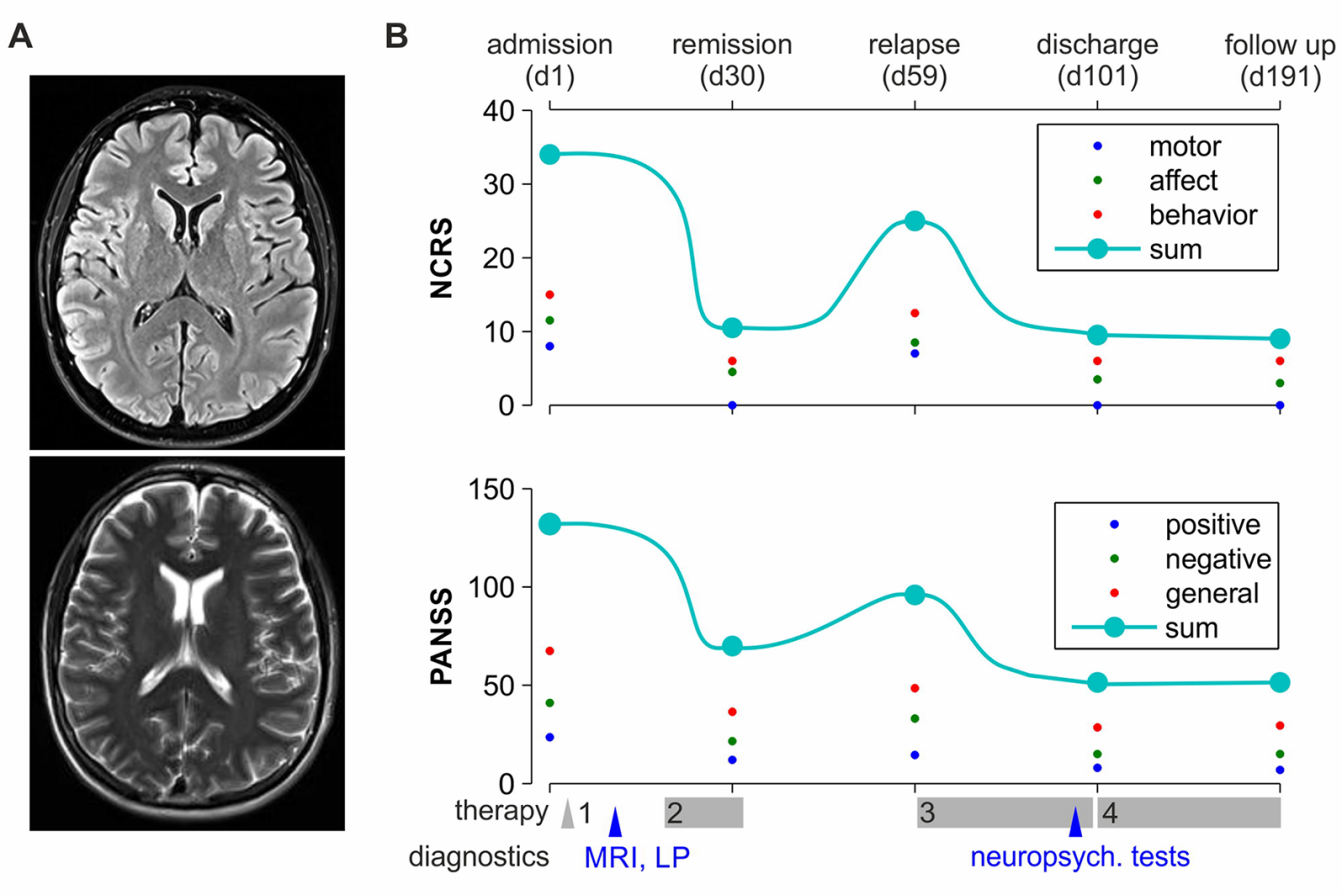
Clinical time course. **(A)** Patient’s MRI, sample horizontal planes (3 mm) at the height of ventricles, taken on a 3-Tesla scanner. Upper image: Turbo-Inversion Recovery-Magnitude (TIRM) sequence with fluid suppression; bottom: T2 blade sequence. **(B)** The time course of NCRS and PANSS scores is depicted on the upper and the lower subplots, respectively. The patient was independently scored at five time points throughout treatment and follow-up (as marked on the upper *x* axis) by the two first authors of the paper; then, the mean score was calculated. Big cyan-filled circles represent the total scores; small red, blue, and green dots represent the subdomains of the tests. Major therapeutic and diagnostic milestones are marked on the bottom *x* axis. Treatment 1–4: 1, enforced medication with lorazepam and isotonic infusions; 2, enforced medication with lorazepam and oral olanzapine 10–15 mg daily; 3, enforced medication with intramuscular olanzapine 300 mg biweekly; 4, voluntary medication with intramuscular olanzapine 300 mg biweekly.

On the ward, the patient initially refused water, food, and medication. Remaining in bed in a rigid posture, appearing confused and anxious, and avoiding eye contact and any kind of communication, he exhibited classic psycho-motoric symptoms of catatonia such as stupor, waxy flexibility, and mutism. The Positive and Negative Syndrome Scale (PANSS) (7) on admission added up to 148 points; the Northoff Catatonia Rating Scale (NCRS) (5) added up to 30 points in total, indicating severe psychosis and catatonia, respectively (see also **Figure 1B**). Due to a sudden state of agitation, the patient had to be temporarily restrained. Initial blood tests, clinical examination, and cranial magnetic resonance tomography (**Figure 1A**) were without significant pathological findings. In particular, no gross abnormalities (e.g., tumor, space-occupying cystic lesion greater than 3 mm, signs of bleeding, contusion, infarction, and major gray or white matter lesions) were found. Due to reduced health conditions of the patient (dehydration, fever, and elevated CreatinKinase) on day 3, treatment with intravenous lorazepam and electrolyte solutions was initiated under physical constraints to prevent malignant catatonia. This led to stabilization of the patient’s state with partial remission of catatonic symptoms. Although some level of communication could be achieved, the patient, however, still refused any kind of treatment and demanded discharge. Therefore, his father was installed as legal guardian; the patient was involuntarily committed to treatment according to German law, and coerced antipsychotic medication with olanzapine and lorazepam was legally approved. Administration of oral olanzapine 20 mg daily over the course of 2 weeks and a subsequent intramuscular injection of 300 mg of its depot formulation resulted in further remission (**Figure 1B**) and allowed for a more in-depth exploration and neuropsychiatric testing.

Five weeks after admission, the patient began to leave his room for dinner and ward rounds if requested. However, he never addressed someone actively. He was fully oriented and did not show any positive psychotic symptoms. Psychomotorically, he was calm and slow. He barely showed emotions, his affect was flat, and his voice and body language were monotonous. Any kind of communication, however, evoked apparent unease in the patient, accompanied by vegetative arousal signs such as sweating and tachycardia. In conversations, he never held eye contact. He still insisted on discharge, stressing out his will to return to his usual daily activities undisturbed by others. The patient admitted having been in critical conditions on admission, but rejected disease-related medical explanations. He refused voluntary medication, repeating that all it required to maintain his health was good food and his daily routine, which mostly comprised playing computer games, watching television, and cooking for himself. He reported an inverted circadian cycle of nightly waking and sleeping from early in the morning to late in the afternoon, so—despite living under one roof—he would rarely interact with his parents and younger sister. He would not leave the house for weeks except for short walks. He did not work and relied financially on his parents who also did all the shopping and housework. He reported not having had any friends or social contacts outside the family for 3 to 4 years. He never had a sexual relationship, albeit being sexually aroused by women, e.g., on television. Asked about his future plans, the patient seemed to be fully convinced that his current living situation would continue unchanged for an indefinite amount of time and considered this positive. When aging and potential mortality of his parents were addressed, he seemed not to be able to conceptualize this possibility. When asked about his parents’ and sister’s ages, he missed it by more than a decade (estimating them younger), albeit being fully oriented and exactly knowing his own age and date of birth.

The patient was born in Germany in a middle-class family. According to his parents, he was a healthy, normal-weight infant without any complications during pregnancy or birth (*via naturalis*). No infections of mother or child were memorable. His motor development was retarded; reportedly, he started to walk at the age of 2. Later, he would have coordinative difficulties to prepare bread or to walk downstairs. The speech development was reportedly unaltered. At the age of 3, he attended kindergarten. After the birth of his younger sister (by 5 years), he would have reacted “properly” and care for her. At the age of 7, he was sent to school. His teacher in the first grade advised the parents to put him in a special school due to his “odd” behavior, but they refused. During kindergarten and school, he was reported to have a hard time finding friends. The patient passed middle school with mediocre grades. According to himself, he did not like a special subject, but was bad at math and reading. He acquired a driver’s license at 18. After school, he finished a full training for a computer technician but did not attend the final exams. According to his parents, this was a breaking point in the patient’s life. He withdrew socially and ceased to adapt to social norms. He was dismissed from an internship at a warehouse due to inability to cope with the social structure at work and disrespect toward supervisors. Until clinical presentation, he had been living in the basement without much contact to the outer world. Interestingly, a stress situation could be described, which might have contributed to the acute psychotic state. The family reported that a renovation took place in the patient’s basement. Therefore, he had to move temporarily upstairs, and his daily routine was disrupted. Asked about this situation, he reported that the inability to withdraw and to follow his routines evoked feelings of anger and fear of change.

We performed a series of neuropsychological tests on the patient. In contrast to the clinical impression and his education, he performed poorly on the Intelligence Structure Test (IST-2000R) IQ test (8), reaching an overall IQ of 74 (**Table 1.1**). However, the patient worked extremely slowly, over-carefully and anxiously, not proceeding adequately, leaving the majority of tasks uncompleted. He showed a substantial lack of motivation and worked only on tasks corresponding to his interests, leaving everything else out. Those items that he completed were solved correctly. Therefore, the results might underrepresent his intelligence. Furthermore, the *Marburger Beurteilungsskala zum Asperger Syndrom* (MBAS) (9), the Structured Clinical Interview for DSM-IV-TR (SCID II) (10), and a facial emotion recognition test were carried out. In the MBAS, the patient reached the cutoff for high-functioning ASD (**Table 1.2**). He could identify all emotions except for anger (**Table 1.4**). The patient underwent Autism Diagnostic Observation Schedule (ADOS-IV) after the partial remission of psychotic symptoms. This module is used with adolescents and adults with fluent speech (11). In this testing, he fulfilled the criteria for ASD and showed deficits mainly in communication and social interaction. The total score of ADOS-IV was 17 points (**Table 1.5**, A4 = 1; A8 = 2; A9 = 2; A10 = 1; B1 = 2; B2 = 2; B6 = 2; B8 = 2; B9 = 1; B10 = 1; B11 = 1; D1 = 1; D2 = 2; D4 = 1; and D5 = 1) and he reached the cutoff criterion for ASD. Autism Diagnostic Ineterview (ADI-R) was not performed. Still, the patient’s parents were interviewed extensively about his development.

**Table 1:** Neuropsychological measures.

1.1 IST-2000R IQ test	
**Domain**	**Score**
Verbal intelligence	67
Numerical intelligence	84
Figurative–spatial intelligence	72
Mean	74
1.2 MBAS	
**Domain**	**Score (cutoff)**
A (Theory of mind, social interaction)	35 (38)
B (Divided attention, facial expression, gestures)	32 (21)
C (Stereotypes and situation-inadequate behavior)	24 (20)
D (Peculiar speech patterns, interests, movements)	23 (16)
Total	114 (103)
Retardation of speech development	Yes
1.3 SCID-II	
**Domain (only domains with reached cutoff listed)**	**Score (cutoff)**
Avoidant	6 (4)
Obsessive–compulsive	5 (4)
Schizoid	4 (4)
Antisocial	3 (3)
1.4 Facial emotion recognition	
**Emotion**	**% correct (latency [s])**
Fear	87 (2.18)
Anger	47 (2.64)
Happiness	100 (2.21)
Neutral	87 (3.51)
Total mean	80 (2.59)
1.5 The Autism Diagnostic Observation Schedule	Score (cutoff)
Total	17 (7)

Continued lack of patient’s therapeutic adherence and legal restrictions resulted in an interruption of therapy with olanzapine depot. Within a few weeks after discontinuation, the patient returned to a mutistic state, mostly refused to communicate, to leave his bed, and to participate in any social activities. He re-expressed high levels of anxiety and psychomotor agitation accompanied by vegetative symptoms. Resumption of forced medication with biweekly intramuscular olanzapine depot led once again to rapid remission. To encourage the patient to engage in social activities, we installed a behavioral plan where participation in ward rounds, ergotherapy, and meals was rewarded by computer time. In cooperation with psychologists and social workers, we strongly involved the parents in the therapeutic process. After multiple extensive psychoeducational sessions, the patient started to accept his biweekly medication. He was discharged in good physical and mental condition after 101 days of treatment (Figure 1B). Currently, he biweekly visits our outpatient office for medication and clinical controls and has been in remission for over a year.

## Discussion

To the best of our knowledge, we present the first case report of an adult male patient with an acute catatonic syndrome on the background of high-functioning ASD. The patient’s clinical presentation and autobiography clearly fulfill the diagnostic criteria of high-functioning ASD. He exhibited profound deficits in socio-emotional behavior and nonverbal communication, had rigid daily routines, and was involved in very few specific social activities. According to his parents, these traits have already been present in early childhood. Despite these psychosocial deficits, he managed to finish middle school, pass the driver’s exam, and receive an Information Technology (IT) education. This is also in line with previous evidence. Although ASD are lifelong neurodevelopmental disorders, the improvement of the autistic core symptoms in the course of the disease is a frequent phenomenon. People with autism and normal intelligence adapt to social and interactional demands as they grow older. This means that the autistic core symptoms might improve across childhood and adolescence (6, 12). However, the occurrence of comorbid psychiatric diseases such as depression, anxiety disorders, or psychoses can lead to a further deterioration of the autistic core symptoms because the compensatory mechanisms no longer function. In our case, the current psychotic exacerbation associated with catatonic syndrome appeared to be triggered by disruption of his daily routines and unusual exposure to social stress and stimuli. Typically triggered by environmental stress, psychotic episodes are a known phenomenon in ASD with prevalence between 4% and 20% (13–15). Catatonic syndrome is also described in ASD with a similar prevalence of about 17% (16). However, we believe that the patient’s biography and clinical course cannot be explained by a single psychotic episode, but rather by the diagnosis of catatonic schizophrenia according to ICD-10 or schizophrenia with catatonia as a specifier according to DSM-5 (17, 18). A clear breaking point can be observed in the patient’s biography, marked by a sudden onset of psychotic symptoms including disorganization, negative symptoms, and psychomotor abnormalities, causing a severe drop in the patient’s social functionality far below his previous level. This is a typical manifestation age of schizophrenia spectrum disorders, which can be followed by an extended prodromal phase without apparent positive symptoms (19). The sudden onset in the young adult patient also makes the diagnosis of schizotypal personality disorder highly unlikely, which usually manifests gradually during adolescence (20). Furthermore, the exacerbation of psychotic symptoms associated with catatonic syndrome upon disruption of antipsychotic therapy in contrast to stable state under medication hints towards catatonic schizophrenia according to ICD-10. In the last two decades, several studies pointed towards possible co-occurrence of both diseases, especially if ASD had been diagnosed during childhood (21). Volkmar et al. (22) found that about 1% of patients with ASD develop schizophrenia spectrum disorders. The prevalence of catatonia in adolescent and young adult ASD patients is described as ∼10% (23). Remarkably, some authors argue that catatonic symptoms and schizophrenia in children with ASD are different manifestations of a single underlying form of brain pathology—a kind of “iron triangle” of symptomatology—rather than three separate illnesses (24). In the last two decades, a growing number of studies have reported ASD with comorbid catatonic syndrome (23, 25–28). However, only two case reports described high-functioning ASD patients exhibiting catatonic syndrome. Ellul and colleagues described a child (29) and Takaoka et al. reported a woman with high-functioning ASD (30). However, the overall risk of catatonic syndrome or schizophrenia in ASD is unknown. The currently reported low prevalence may be due to the fact that ASD prior to onset of schizophrenia may be strongly unrecognized (31). Indeed, positive, negative, and motor symptoms may occur in both conditions and therefore present a diagnostic challenge (32–34). Intriguingly, according to the results of a parental interview, about 50% of patients with schizophrenia spectrum disorders retrospectively fulfilled ASD criteria (35). This staggeringly high co-occurrence is not surprising if one takes into account the pathophysiological similarities between these conditions. Although there has been an increase in ASD diagnoses in recent years, the increasing prevalence rates are mainly related to children or adolescents with early childhood autism and mental retardation. ASD patients with normal intelligence often become manifest in adulthood by a different psychiatric diagnosis as their life circumstances or needs change. Along with clinical overlaps, both schizophrenia and ASD are considered as neurodevelopmental disorders and manifold overlap in their genetics, pathophysiology, and symptoms to an extent that some authors discuss them as entities on one disease spectrum (36). Thus, pregnancy-related complications were identified as significant risk factors for both conditions (37). Common genetic risk factors for both ASD and schizophrenia include genes encoding neurotrophic factors such as neurexin-1 (38) and synaptic proteins such as PSD-95 (39) and copy number variations in several genomic regions (e.g., 1q21, 16p11.2, and 22q11) regulating synaptic structure and function (40). These mutations are also successfully employed to model these diseases in rodents (41, 42). Both conditions furthermore share various pathophysiological endophenotypes including altered evoked auditory potentials and sensorimotor gating deficits (40, 43), imbalances in the excitatory–inhibitory activity (44), and overlapping findings in imaging studies such as altered gray matter volumes within the limbic–basal ganglia loop system (45, 46). In particular, it has been shown that basal ganglia are important structures in the pathogenesis of ASD, because of their crucial role in motor and cognitive functioning (47). Thorough examination enables the clinical practitioner to distinguish between these conditions in unclear cases such as the one presented. Neuropsychologically, patients with ASD were shown to be predominantly impaired in executive functioning tasks such as planning/organization and flexibility of thought, whereas patients with schizophrenia had impairments in a wide range of intellectual abilities (48). Furthermore, patients with schizophrenia showed significantly larger motor coordination deficits (neurological soft signs) as compared to ASD patients (46). Additionally, the patient’s history, especially careful parental anamnesis, can provide crucial clues to differentiate between ASD and schizophrenia or both even in an adult, unknown patient with no history of disease.

## Concluding remarks

In unknown adult patients presenting with catatonic syndrome, ASD need to be considered among potential differential diagnoses. Physicians need to be aware that both conditions may co-occur in one individual. Despite diagnostic and therapeutic challenges, thorough examination and meticulous anamnesis can lead to correct diagnosis and facilitate treatment. Even when typical autistic features are missing due to severe catatonic syndrome, ASD should be considered, even early in the diagnostic process in order to correctly classify the interactional difficulties with such patients. Elicitation of incongruous or unusual psychopathological findings should be followed up by careful psychometric and neuropsychological examination as well as by case history through a third party (e.g., parents or siblings).

### Clinical Warning Signs That Enable Earlier Diagnosis of Autism Spectrum Disorders, Therapy, and Better Prognosis

- Behavioral features such as repetitive and/or stereotyped patterns of movement, clumsy gait, poor muscle tone, imbalance, impairment of motor skills, abnormal involuntary movements, monotonous voice and body language, mutism, and negativism
- Sudden onset of catatonic and/or psychotic symptoms following stressful life events or sensory overload (CAVE: hyper- and/or hyposensitivity to external stimuli)
- Difficulties to maintain a conversation with others and orienting to people in a social environment due to impairments in social interaction, language, and related cognitive skills
- Limitations with maintaining turn-taking in interactions with others and difficulties in responding to bids for interaction often leading to vegetative arousal signs such as sweating, tachycardia, and internal unrest

## Consent for publication

The father of the patient gave his written consent for information about his son to be published in a scientific journal. He understood that the information will be published without his son’s name attached, but that full anonymity cannot be guaranteed. He understood that the text and MRI images published in the article will be freely available on the Internet and may be seen by the general public. The MRI images and text may also appear on other websites or in print and may be translated into other languages or used for commercial purposes. The father has been offered the opportunity to read the manuscript. A copy of the written consent is available for review by the editor of this journal.

## Ethics Statement

Not applicable. Written informed consent was obtained from the patient and his legal guardian for publication of this case report and any accompanying images. A copy of the written consent is available for review by the editor of this journal.

## Author Contributions

DHe and CT equally contributed to this work. DHe and CT performed the treatment, neuropsychological tests, took the patient history and third-party anamnesis, and wrote the paper. PG supervised all steps of diagnoses and treatment and wrote the paper. DHi supervised neuropsychological testing and wrote the paper.

## Funding

This work was supported by the Physician Scientist Program of the Medical Faculty of the Heidelberg University. We acknowledge financial support by Deutsche Forschungsgemeinschaft within the funding program Open Access Publishing, by the Baden-Württemberg Ministry of Science, Research and the Arts and by Ruprecht-Karls-Universität of Heidelberg.

## Conflict of Interest Statement

The authors declare that the research was conducted in the absence of any commercial or financial relationships that could be construed as a potential conflict of interest.
